# Association between the ABCA1 rs1800977 polymorphism and susceptibility to type 2 diabetes mellitus in a Chinese Han population

**DOI:** 10.1042/BSR20171632

**Published:** 2018-03-09

**Authors:** Chenyi Li, Danjun Fan

**Affiliations:** Department of Medical Care for Cadres, Zhejiang Hospital, 12 Lingyin Road, Hangzhou, Zhejiang, China

**Keywords:** ABCA1, Chinese population, polymorphism, type 2 diabetes mellitus

## Abstract

ATP-binding cassette transporter A1 (ABCA1) is associated with serum high-density lipoprotein (HDL) levels. Several studies have demonstrated that individuals with a high HDL cholesterol level have a reduced risk of incident type 2 diabetes mellitus (T2DM). Therefore, we conducted a case–control study including 508 T2DM patients and 614 controls to explore the association between the ABCA1 rs1800977 polymorphism and T2DM risk in a Chinese Han population. Genotyping was performed using matrix-assisted laser desorption/lionization time-of-flight mass spectrometry. Our results indicate that the TT genotype of the rs1800977 polymorphism was associated with a decreased risk of T2DM compared to the CC genotype. The T allele of the rs1800977 polymorphism was also related with a decreased risk of T2DM. There was no significant association between clinical parameters (HDL, low-density lipoprotein, cholesterol, body mass index, and age) and rs1800977 polymorphism genotypes. In conclusion, the ABCA1 rs1800977 polymorphism may contribute to the development of T2DM. However, larger studies with more diverse ethnic populations are needed to confirm these results.

## Introduction

The prevalence of type 2 diabetes mellitus (T2DM), one of the most common chronic conditions worldwide, has increased continuously primarily due to the obesity epidemic [1]. It is estimated that 380 million people will have T2DM and 418 million people will have impaired glucose tolerance by 2025 [2]. However, the etiology of T2DM is poorly understood. Genome-wide association studies have identified more than 70 loci associated with T2DM [3]. The roles of genetic risk factors in the pathogenesis of T2DM have attracted much attention [4].

The ATP-binding cassette transporter A1 (ABCA1) gene is located at chromosome 9q31.1 and mediates the secretion of cellular-free cholesterol and phospholipids to an extracellular acceptor, apolipoprotein AI, to form nascent high-density lipoprotein (HDL) [5]. Emerging lines of evidence have demonstrated that the transcriptional up-regulation of ABCA1 promotes HDL formation and reverse cholesterol transport [6]. The absence of ABCA1 may contribute to glucose intolerance and cholesterol accumulation within the beta cell plasma membrane, which plays important roles in the pathogenesis of T2DM [7]. Ren et al. [8] reported a positive association between HDL cholesterol and insulin resistance in Chinese patients with newly diagnosed T2DM, while Hwang et al. [9] confirmed that individuals with a higher HDL cholesterol level were at a lower risk of incident T2DM. We hypothesized that ABCA1 is involved in the etiology of T2DM by regulating the expression of HDL.

Porchay et al. [10] first found that the T allele of the rs1800977 polymorphism was associated with higher HDL cholesterol levels in normal-weight men (body mass index [BMI] < 25 kg/m^2^). Several studies attached importance to the role of the ABCA1 rs1800977 polymorphism in the risk of T2DM, but with conflicting results [11–13]. Haghvirdizadeh et al. [13] found that the ABCA1 rs1800977 polymorphism increased the risk of T2DM among Malaysians. However, two studies indicated that this single nucleotide polymorphism (SNP) was associated with a decreased risk of T2DM in Saudi and Turkish populations [11,12]. However, no study has investigated an association between the ABCA1 rs1800977 polymorphism and T2DM risk in a Chinese Han population. Therefore, we conducted a hospital-based case–control study (508 cases and 614 controls) of a Chinese Han population from Zhejiang province to evaluate whether the rs1800977 polymorphism of the ABCA1 gene confers susceptibility to T2DM.

## Materials and methods

### Study subjects

A total of 508 T2DM patients were included in this study. The patients were admitted to Zhejiang Hospital between October 2013 and December 2016. The inclusion criteria for the controls were: individuals with a fasting blood glucose level ≥ 126 mg/dl or a 2-h postprandial blood glucose level ≥ 200 mg/dl or a glycated hemoglobin level > 6.5% and without a family history of diabetes [14]. Cases with type 1 diabetes, acute infections, cancer, myocardial infarction, or kidney disease were excluded from this study. A total of 614 age- and sex-matched non-diabetic controls were recruited from the medical center of Zhejiang Hospital. Subjects who had been diagnosed with cancer, type 1 diabetes, a genetic malformation, or pregnancy were excluded from this study. None of the subjects were receiving any drugs such as antidiabetic, antihypertensive, or hypolipidemic drugs to abolish the potential confounding effects of these medications. A full clinical examination, including blood pressure and anthropometric measurements involving BMI, was performed. The study was approved by the research ethnical committee of Zhejiang Hospital and followed the Declaration of Helsinki. Written informed consent was obtained from all study participants.

### Blood sampling and genotyping

Peripheral blood (2 ml) was collected from each subject using test tubes containing ethylene diamine tetraacetic acid. Genomic DNA was extracted from peripheral blood using a QIAamp DNA Blood Mini Kit (Qiagen, Hilden, Germany) according to the manufacturer’s instructions. DNA samples were tested for quality and concentration using an ultraviolet spectrophotometer and stored at −20°C. High-throughput matrix-assisted laser desorption/lionization time-of-flight mass spectrometry (Genesky Biotechnologies Inc., Shanghai, China) technology was applied for genotyping. Primers specific for the rs1800977 polymorphism were used for polymerase chain reaction (PCR). The sequences of the primers were as follows: 5′-CAG CGC TTC CCG CGC GTC TTA-3′ (forward) and 5′-CCA CTC ACT CTC GTC CGC AAT TAC-3′ (reverse). Amplification was carried out in a DNA thermal cycler with 33 cycles of denaturation at 94°C for 1 min, annealing at 60°C for 1 min, and extension at 72°C for 2 min. The PCR products were then sequenced using MassARRAY 4.0 (Agena Bioscience, San Diego, CA, U.S.A.).

### Statistical analysis

All statistical analyses were performed using Stata 11.0 software (StataCorp, College Station, TX, U.S.A.). An analysis of variance and Student’s *t*test was used to compare the clinical parameters between cases and controls and to compare the clinical parameters of the controls with different genotypes. The proportions of groups were compared by the χ^2^ test. The odds ratio (OR) and 95% confidence interval (CI) were calculated to evaluate the association between the ABCA1 rs1800977 polymorphism and T2DM risk by logistic analysis. The threshold for significance was set at *P*<0.05.

## Results

### Subject characteristics

In the present study, 508 T2DM patients and 614 healthy age- and sex-matched controls (*P*=0.915 and *P*=0.523, respectively) were enrolled. The demographic and medical data in the case and control groups are shown in Table 1. The mean age was 55.27 years for T2DM patients and 55.20 years for the control subjects. The values of hypertension, fasting blood glucose, BMI, cholesterol, and low-density lipoprotein (LDL) were higher in the patient group than in the control group (*P* 005). However, there was no significant difference between the two groups concerning HDL cholesterol.

**Table 1:** Patient demographics and risk factors in type 2 diabetes mellitus

Variable	Cases (*n* = 508)	Controls (*n* = 614)	*P*
Age (years)	55.27 ± 13.30	55.20 ± 10.21	0.915
Female/male	208/300	263/351	0.523
Hypertension (no/yes)	297/211	508/106	<0.001
BMI (kg/m^2^)	26.10 ± 4.73	25.42 ± 4.62	0.016
Fasting blood glucose (mmol/l)	9.06 ± 2.01	5.83 ± 1.62	<0.001
Total cholesterol (mmol/l)	4.85 ± 0.85	4.34 ± 0.94	<0.001
HDL (mmol/l)	1.17 ± 0.38	1.13 ± 0.40	0.080
LDL (mmol/l)	3.18 ± 1.06	2.85 ± 0.69	<0.001

### Association analysis

The genotype frequencies of the ABCA1 rs1800977 polymorphism in the cases and controls are summarized in Table 2. The TT genotype was associated with a decreased risk of T2DM in recessive and homozygous models (TT vs. CC, OR = 0.50, 95% CI = 0.34–0.74, *P*=0.001). In addition, the T allele decreased the risk of T2DM (*P*=0.003). No relationship between genotypes of the ABCA1 rs1800977 polymorphism and clinical characteristics (age, fasting blood glucose, total cholesterol, HDL, LDL, and BMI) was identified (Table 3).

**Table 2:** Logistic regression analysis of associations between ABCA1 rs1800977 polymorphism and risk of type 2 diabetes mellitus

Genotype	Cases* (*n* = 508)		Controls* (*n* = 614)		OR (95% CI)	*P*
	*n*	%	*n*	%		
CT vs. CC	201/258	39.6/50.8	223/285	36.3/46.4	1.00 (0.77–1.28)	0.973
TT vs. CC	46/258	9.1/50.8	101/285	16.4/46.4	0.50 (0.34–0.74)	0.001
CT+TT vs. CC	247/258	48.6/50.8	324/285	52.8/46.4	0.84 (0.67–1.07)	0.154
TT vs. CT+CC	46/459	9.1/90.4	101/508	16.4/82.7	0.50 (0.35–0.73)	<0.001
T vs. C	293/717	28.8/70.6	425/793	34.6/64.6	0.76 (0.64–0.91)	0.003

Bold values are statistically significant (*P*<0.05).

* The genotyping was successful in 505 cases and 609 controls.

**Table 3:** The clinical and biochemical characteristics of ABCA1 rs1800977 polymorphism among two groups

	Patients (*N* = 505)				Controls (*N* = 614)			
	CC (*N* = 258)	CT (*N* = 201)	TT (*N* = 46)	*P*	CC (*N* = 285)	CT (*N* = 223)	TT (*N* = 101)	*P*
Age (years)	55.21 ± 13.32	54.35 ± 13.57	59.83 ± 11.59	0.090	55.08 ± 10.37	55.32 ± 9.83	55.21 ± 10.75	0.989
BMI (kg/m^2^)	26.23 ± 4.70	25.95 ± 4.80	26.10 ± 4.83	0.887	25.34 ± 4.63	25.55 ± 4.61	25.32 ± 4.67	0.895
Fasting blood glucose (mmol/l)	9.20 ± 1.91	8.91 ± 2.06	8.86 ± 2.34	0.354	5.76 ± 1.57	5.86 ± 1.69	5.98 ± 1.59	0.479
Total cholesterol (mmol/l)	4.89 ± 0.85	4.80 ± 0.83	4.86 ± 0.85	0.438	4.33 ± 0.92	4.33 ± 0.96	4.39 ± 0.95	0.212
HDL (mmol/l)	1.19 ± 0.39	1.16 ± 0.38	1.14 ± 0.32	0.879	1.11 ± 0.39	1.13 ± 0.39	1.16 ± 0.43	0.582
LDL (mmol/l)	3.17 ± 1.11	3.21 ± 1.01	3.04 ± 0.91	0.645	2.81 ± 0.69	2.85 ± 0.68	2.97 ± 0.73	0.218

## Discussion

In this study, the ABCA1 rs1800977 polymorphism correlated with a decreased risk of T2DM in a Chinese Han population. However, no significant association was observed between genotypes of the rs1800977 polymorphism and lipid levels among the two groups.

Cholesterol accumulation may be a risk factor for glucose intolerance and diabetes [15]. ABCA1, a key molecule in cholesterol homeostasis, plays an important role in clearing excess cholesterol from macrophages [16]. Carriers of loss-of-function mutations in ABCA1 exhibit impaired insulin secretion without insulin resistance [17]. Moreover, the expression and protein concentration of ABCA1 in leukocytes, as well as ABCA1 function in cultured skin fibroblasts, are reduced in T2DM [18]. The T allele of the rs1800977 polymorphism in ABCA1 was reported to be associated with higher HDL cholesterol levels in normal-weight men (BMI < 25 kg/m^2^) [10]. HDL cholesterol is inversely associated with the incidence of T2DM [9].

Several recent studies have focused on the relationship between the ABCA1 rs1800977 polymorphism and T2DM risk. In 2012, Ergen et al. [12] conducted a hospital-based case–control study (107 patients and 50 controls) to explore an association between the ABCA1 rs1800977 polymorphism and lipid levels in Turkish T2DM patients. They found that the T allele and TT genotype of the rs1800977 polymorphism were associated with a reduced risk of T2DM [12]. However, there was no significant association between genotype and lipid concentration [12]. Subsequently, Alharbi et al. [11] attempted to replicate this finding in a Saudi population with a total of 376 cases and 380 controls. The T allele of the rs1800977 polymorphism was identified as a protective factor against T2DM [11]. No relationship between ABCA1 rs1800977 genotypes and lipid profiles was observed [11]. However, Haghvirdizadeh et al. [13] reported that this SNP increased the risk of T2DM among Malaysians. The clinical and biochemical characteristics of this polymorphism did not reveal any differences between the case and control groups [13]. Remarkably, this study consisted of limited sample sizes (164 cases and 165 controls). Therefore, we cannot exclude the possibility that the positive findings of the above studies with smaller sample sizes were due to chance. Our results indicate that TT genotype carriers had a lower risk of T2DM than those who carried the CC genotype in a Chinese Han population with 508 cases and 614 controls. To the best of our knowledge, this is the first study to investigate an association between the ABCA1 rs1800977 polymorphism and T2DM risk. Moreover, we found that the T allele is a protective factor for T2DM, in contrast to the study conducted by Haghvirdizadeh et al. [13]. We did not uncover positive findings between genotypes of the rs1800977 polymorphism and lipid concentration (including total cholesterol, HDL, and LDL), in accordance with previous studies. In summary, clinical heterogeneity, the inclusion of different ethnic populations, and differences in sample size may have contributed to the disparate results of previous studies and the current study. To overcome these limitations and reduce the possibility of false-positives, a meta-analysis for this SNP is needed to derive a more precise estimation of the effects of the ABCA1 rs1800977 polymorphism on T2DM.

Several limitations merit careful consideration. First, selection bias was unavoidable because all participants were from the same hospital and from a Chinese Han population. Second, we only explored one polymorphism of the ABCA1 gene, which may prohibit the comprehensive investigation between ABCA1 gene polymorphisms and T2DM risk. Third, we did not carry out a gene–environment interaction analysis due to a lack of relevant information. Finally, the sample size was moderate; thus, this study may be underpowered.

In conclusion, the ABCA1 rs1800977 polymorphism was associated with a reduced risk of T2DM in a Chinese Han population. Larger studies are warranted to determine whether the ABCA1 rs1800977 polymorphism confers susceptibility to T2DM.

## Abbreviations

ABCA1: ATP-binding cassette transporter A1
BMI: body mass index
CI: confidence interval
HDL: high-density lipoprotein
LDL: low-density lipoprotein
OR: odds ratio
SNP: single nucleotide polymorphism
T2DM: type 2 diabetes mellitus
